# Amalgam

**Published:** 1898-05

**Authors:** 


ARTICLE VIII.

### AMALGAM.

### A DISCUSSION IN ALABAMA SOCIETY.

Dr. R. B. Adair, in *Dental Weekly*, advocates the filling of the teeth—posterior to the first bicuspid—with amalgam. The observance of this practice insuring the saving of more teeth and conserving the strength and time of the operator.

The disrepute in which amalgam is held by some, he says, is largely caused by the hasty and slovenly manner of its introduction. If the same preparation of cavities, and careful manipulation were observed as when gold is used, the essayist predicted results that would challenge the admiration and endorsement of even the most partisan gold man.

Dr. Brosius.—Too large pieces of amalgam should not be placed in a cavity. The first piece in a large cavity should be quite soft, and the successive pieces should be harder as the filling grows to completion, finishing with that which is quite hard. This makes a filling in which the mercury is more thoroughly diffused throughout the mass.

Dr. Rosser.—In the first years of his practice considered that in proportion as he used amalgam the more harm he was doing his patients. Now he is sure his patients would have been better served if he had used more amalgam. Cavities for amalgam should he prepared with as much care as if gold were to be used. Lack of carefulness in detail is the cause of many failures. Each filling should be finished at a second setting. Be careful about overlapping edges. Cement should be used as a base in all large cavities. If two approximate fillings are to be made, put in one at a time and finish it before filling the other.

Dr. Jewett.—Get it out of the patient's mind that because you are using amalgam it must be a cheap filling. Show them that it requires skill and judgment to make a good amalgam filling. The cheap John idea is what does the damage in many instances. It is not an oxid that forms on it, but a sulphid, and that blackens it.

Dr. Chappel.—I am an advocate of amalgam, but care must be taken in both the preparation of the cavity and the introduction of the filling. The rotary motion for packing is not so good as the direct pressure.

Dr. Frank Smith.—Amalgam has been a puzzle to me. I practiced ten years before using it. Too much of it is used. The best results are to be had by using it as a veneer over cement or tin foil. Fill a cavity nearly full with tin and finish with amalgam, which gives a harder surface than tin alone.

Dr. Browne uses a matrix of mica, which is left in till the final finish.

Dr. W. E. Walker.—The matrix of Dr. Browne will not do. Too much overhanging amalgam will be left to be finished off. It is more difficult to finish amalgam at the cervical margin than gold. The best matrix is made of German silver bent to the contour and adapted well at the cervix. Two wedges, one from labial and one from the inner side can be pressed in to hold the matrix right against the wall. The double wedges can be easily removed.—*Dental Weekly*.

